# Nephroprotective and antioxidant properties of a novel herbal extract combination in a high-fat diet and alloxan-induced kidney injury in rats

**DOI:** 10.5455/javar.2026.m1020

**Published:** 2026-03-13

**Authors:** OK Yulizal, Darmadi Darmadi, OK Ikram Alchair, Encik Nazifa Zanzabilla Putri, Wa Amalia Zahiah, Tesalonika Apmarda Simarmata

**Affiliations:** 1 Department of Internal Medicine, Faculty of Medicine and Dentistry, Universitas Prima Indonesia, Medan 20118, Indonesia; 2 Department of Internal Medicine, Faculty of Medicine, Universitas Sumatera Utara, Medan 20155, Indonesia; 3 Faculty of Medicine, Malikussaleh University, Lhokseumawe 24351, Aceh, Indonesia; 4 Faculty of Dentistry, Universitas Sumatera Utara, Medan 20155, Indonesia; 5 Faculty of Medicine and Dentistry, Universitas Prima Indonesia, Medan 20118, Indonesia

**Keywords:** Cystatin C, herbal medicine, nephroprotection, oxidative stress, superoxide dismutase

## Abstract

**Objectives:** The present study investigated the therapeutic effects of a standardized capsule combination consisting of *Channa striata, Phyllanthus niruri* L., and *Curcuma xanthorrhiza* extracts (CCE) on biomarkers of kidney function, antioxidant status, and kidney histopathology in a high-fat diet (HFD) and alloxan-induced diabetic/nephrotoxic rat model.

**Materials and Methods:** Male Wistar rats (*n* = 24) were divided into 4 groups: Control; HFD + alloxan treatment (diabetics/nephrotoxic); diabetics/nephrotoxic + CCE (49.5 mg/kg BW); diabetic/nephrotoxic + CCE + pioglitazone treatment group (1.35 mg/kg BW). Serum Cystatin C, superoxide dismutase (SOD) activity, and kidney histopathology were assessed at Day 60.

**Results:** Model induction markedly increased blood glucose, HbA1c, and serum Cystatin C levels and decreased SOD activity (*p* < 0.001 vs controls). CCE significantly attenuated cystatin C by decreasing Cystatin C by 18.2% (*p* < 0.001) and restored SOD activity to 94.0% of control (*p* < 0.001). Histopathology showed marked improvements in glomerular hypertrophy, tubular injury, vascular congestion, and inflammatory infiltration (*p* < 0.001 vs model). CCE + pioglitazone showed comparable biochemical trends and enhanced histological preservation.

**Conclusions:** CCE combination significantly attenuated diabetic/nephrotoxic-induced kidney injury by improving antioxidant defense and preserving kidney structure. This suggests therapeutic potential for managing metabolic kidney complications.

## 1. Introduction

Non-alcoholic steatohepatitis (NASH) is an advanced stage of non-alcoholic fatty liver disease (NAFLD), including hepatic steatosis, inflammation, and fibrosis, leading to cirrhosis and hepatocellular carcinoma [1, 2, 3]. Indeed, recent epidemiologic data suggested that NASH affects 3–5% of the global population, but with an estimated prevalence rate reaching up to as high as 20–30% of those obese and/or type 2 diabetes cases [2, 4]. This growing disease burden is similar to the global epidemic of metabolic syndrome, and heightens NASH’s role as a leading cause of chronic liver disease and liver-related mortality [3, 4]. In addition to its main hepatic phenotype, NASH produces several systemic disorders, including kidney injury in 20–40% of patients by similar pathophysiology mechanisms [5, 6].

The pathogenesis of these two abnormalities (NASH and kidney injury) is interconnected through multiple ways; mainly, they include oxidative stress, chronic inflammation, and metabolic derangement [5, 6, 8]. Hepatic steatosis can lead to excessive production of reactive oxygen species (ROS) when the host antioxidant defense is overwhelmed, resulting in systemic oxidant stress [7, 8, 9, 10]. The oxidative attack is not exclusive to hepatic tissues; it can also damage the kidneys through lipid peroxidation, protein oxidation, and nucleic acid damage [7, 10]. The first-line antioxidant enzyme, superoxide dismutase (SOD), is extensively reduced in NASH, leading to impairment of cell defence against oxidative stress [9, 11, 12].

Concurrently, other inflammatory molecules originating from steatotic liver tissues, such as tumor necrosis factor-alpha (*TNF-α*) and interleukin-6 (*IL-6*), have also been proven to induce the glomerular and tubular damage [13, 14]. These actions can lead to the detection of alterations in kidney markers, such as Cystatin C, a pivotal early indicator of kidney injury and an alternative to standard creatinine measures because it is independent of muscle mass, age, and dietary protein intake [15, 16, 17].

There is only a limited number of therapeutic options for NASH available today, and therapy ranges from lifestyle change to pioglitazone or vitamin E, the former being used off-label without significant evidence on clinical response and safety [18, 19, 20]. Due to the lack of specific targeted drugs for kidney injury related to NASH or metabolic disorders, interest has been drawn to natural herbal products with hepatoprotective and nephroprotective activities [21, 22]. Traditional natural medicines, due to their multitarget mechanisms, such as antioxidant activation [22], anti-inflammatory activity [23], and metabolic regulation [24], have demonstrated potential therapeutic activity. Among them, *Channa striata* (snakehead fish extract) [25], *Phyllanthus niruri* L. (stonebreaker) [26, 27], and *Curcuma xanthorrhiza* (Javanese turmeric) [28, 29] are several agents that have been found to exert preclinical evidence for their hepatoprotective as well as nephroprotective properties.

*Channa striata* contains bioactive compounds, such as albumin, amino acids, and fatty acids, which support tissue regeneration/wound healing and possess antioxidant activity [25, 26]. Lignans, flavonoids, and phenolic compounds present in *Phyllanthus niruri* L. are known to harbor hepatoprotective, nephroprotective, and antioxidant potential mediated (*Nrf2* pathway) through the modulation of oxidative stress pathways together with inflammatory cascades [27, 28]. *Curcuma xanthorrhiza* was rich in curcuminoids (through inhibition of *NF-κB*) and essential oils. It was previously known to have strong anti-inflammatory and antioxidative effects, as well as to improve metabolic profiles [29, 30]. While each of these 3 herbs has been separately reported to exhibit hepatoprotective effects, no experimental data are available regarding the combinatorial effect in a standardized preparation of such formulas on kidney injury under NASH-like metabolic disorder conditions. These complementary actions may collectively protect the kidney from oxidative and inflammatory insults in a diabetic/nephrotoxic environment.

The present study focuses on kidney injury occurring in a high-fat diet (HFD) and alloxan-induced diabetic/nephrotoxic rat model. It reproduces key metabolic and oxidative features often seen in patients with NASH and type 2 diabetes. The HFD and alloxan model mimics diabetic nephropathy with hyperglycemia, oxidative stress, and direct tubular toxicity, commonly used for nephroprotective screening despite limitations (e.g., chemical confounder vs pure NASH) [31, 32, 33]. The HFD component promotes hepatic steatosis, dyslipidemia, and insulin resistance, while alloxan induces hyperglycemia and direct tubular toxicity [31, 32, 34]. However, in this study, we did not conduct liver histology or biochemistry analyses for hepatic steatosis, inflammation, or fibrosis. This model should be considered a metabolic/diabetic nephrotoxic rather than a histologically validated NASH model.

Consequently, kidney injury in this setting is more accurately described as arising from combined diabetic and nephrotoxic mechanisms under NASH-like metabolic conditions.

Therefore, the aim of this study was to assess the therapeutic potential of the standardized capsule combination extract (CCE) containing *Channa striata, Phyllanthus niruri* L., and *Curcuma xanthorrhiza* in an HFD- and alloxan-induced diabetic/nephrotoxic rat model, as a NASH-like metabolic condition. We hypothesized that combining these three herbal compounds might improve kidney function by restoring antioxidant levels and reducing kidney injury biomarkers. The specific aims of this study were to assess serum Cystatin C levels, SOD activity, and kidney histopathological changes after CCE and to compare them with those of pioglitazone combination treatment [18, 20]. These findings are expected to provide preliminary insights into the potential role of this herbal combination in mitigating kidney complications associated with metabolic and NASH-related conditions.

## 2. Materials and Methods

### 2.1. Ethical approval

The experimental protocol was approved by the Health Research Ethics Committee of Universitas Prima Indonesia, Medan, Indonesia (Approval No: 046/KEPK/UNPRI/III/2024; June 15^th^ 2024), and it conformed to international standards for animal studies.

### 2.2. Study design and sample size

This study included an experimental research design with a posttest-only control group and simple random sampling. The sample size calculation was performed with the G*Power software (version 3.1.9.7), setting *α* = 0.05, power (1–β) = 80% and effect size (*f* = 0.50) based on preliminary data and relevant related studies [35]. This estimation led to a minimum of 5 animals per group. Initially, 6 animals per group were selected to account for anticipated animal losses during the 60-day study period.

### 2.3. Experimental animal and housing conditions

Twenty-four Wistar rats (*Rattus norvegicus*), male, body weights ranging from 150–200 gm, and aged 8–10 weeks, were purchased from Ellio Laboratory Company, Medan, Indonesia. We selected male rats to exclude possible confounding effects of hormonal changes on metabolic and oxidative stress parameters [36]. Animals were housed in standard polycarbonate cages (3 rats/cage) at 22 ± 2°C, 50–60% humidity, and a 12 h light/dark cycle (06:00 ON/18:00 OFF). All animals were acclimated for 7 days prior to adaptation and experimental manipulations to reduce the confounding effects of stress-related factors [37].

### 2.4. HFD and alloxan induced diabetic/nephrotoxic

A high-fat diet (HFD) in conjunction with alloxan was used to induce a diabetic/nephrotoxic model. This procedure produces key metabolic and oxidative features often associated with NASH and its renal complications. This was generated with a validated two-hit model incorporating diet manipulation and chemical induction [31, 32, 33]. The dietary part was carried out with an HFD, which consisted of 60% fat, 20% protein, and 20% carbohydrate, provided orally through a gavage tube every day for 4 weeks (day 8-day 35). This includes being lard as 3 gm/200 gm body weight (BW) of lard and 2 gm/200 gm BW of duck egg yolk. This diet promotes hepatic steatosis, dyslipidemia, and insulin resistance as occurs in NASH [31, 32].

On the 29^th^ day, animals were injected with a single dose of alloxan monohydrate (Sigma-Aldrich, USA) in sterile normal saline (0.9% NaCl w/v) at 100 mg/kg BW [33].

Previous articles reported that this dual-hit rat model reproduces the main features of kidney injury in the HFD/alloxan-induced diabetic nephrotoxic model [31, 32, 33]. This induction was confirmed on day 30 (before the start of treatments) by determining fasting blood glucose concentration. Animals successfully induced were those with blood glucose ≥ 200 mg/dl and clinical signs of metabolic failure (polydipsia, polyuria) in the following days [33]. However, hepatic NASH characteristics (steatosis, inflammation, and fibrosis) were not confirmed by liver histology or biochemistry; thus, kidney injury is more dependent on diabetes plus alloxan nephrotoxicity than pure NASH pathophysiology.

### 2.5. Preparation and characterization of capsule combination extract (CCE)

The standardized capsule combination extract (CCE) was obtained commercially as a ready-made product: Fitbumino^®^ capsules (PT. Akar Rimba Nusantara, Jakarta, Indonesia). Each capsule contained 500 mg of *Channa striata* extract (standardized to more than 15% protein content), 400 mg of *Phyllanthus niruri* L. extract (standardized to more than 2% phyllanthin and hypophyllanthin), and 50 mg of *Curcuma xanthorrhiza* extract (standardized to more than 5% curcuminoid content) [25, 27, 29]. The extracts were processed by the manufacturer using specific extraction methods. This produces a validated protein-rich aqueous extraction product for *Channa striata* (protein-rich extract), ethanol (70% v/v) extraction for *Phyllanthus niruri*, and ethanol (96% v/v) extraction for *Curcuma xanthorrhiza* [25, 27, 29]. For oral gavage, capsule contents were dissolved in 0.5% Sodium carboxymethyl cellulose (Na–CMC; Sigma–Aldrich, USA) to achieve a dose of 9.9 mg/200 gm BW, equivalent to approximately 49.5 mg/kg BW [38]. This dose was based on its traditional use, previous preclinical studies that showed the safety and efficacy of these individual components, and allometric scaling from human equivalent doses [25, 26, 27, 28, 29, 30, 38]. The suspension was freshly prepared once a day and given at 1 ml per oral gavage.

### 2.6. Experimental groups and treatment protocol

All rats were fed (*ad libitum*) with standard laboratory chow (PT Japfa Comfeed Indonesia) and tap water. Animals were randomized into 4 groups by random number list seven days before the experiment [37]:

- Group-1 (Control, *n* = 6): The healthy animals were fed on standard laboratory chow for the entire duration of the experiment (day 1 to day 60). Orally treated with 1 ml 0.5% Na–CMC vehicle once per day from day 31 to day 60.
- Group-2 (Diabetic/nephrotoxic (HFD + alloxan), *n* = 6): Animals received an HFD (days 8–35) and an alloxan injection (day 29). Administered with 1 ml/day of vehicle alone (0.5% Na-CMC) from day 31 to day 60 as a diabetic/nephrotoxic untreated control.
- Group-3 (Diabetic/nephrotoxic + CCE, *n* = 6): Animals were treated under an HFD-alloxan-induced diabetic/nephrotoxic condition (as Group-2 2). They were treated with CCE (9.9 mg/200 gm BW), which dissolved in 1 ml of 0.5% Na-CMC, by oral gavage once daily from day 31 to day 60.
- Group-4 (Diabetic/nephrotoxic + CCE + Pioglitazone, *n* = 6): Animals as in Group-3 + treated with Pioglitazone. Administered both CCE (9.9 mg/200 gm BW) and pioglitazone. Pioglitazone (PT. Dexa Medica, Indonesia; 15 mg/tablet) was administered at a dose of 0.27 mg/200 gm BW (~1.35 mg/kg BW), suspended in 1 ml of 0.5% Na-CMC and given orally daily from day 31 to day 60 [18, 20]. This relatively low chronic dose was selected to approximate human-equivalent exposure for long-term tolerability [39, 40, 41, 42].

All interventions occurred between 08:00 and 10:00 in the morning, to reduce circadian differences [37]. During this study, the body weight was controlled once a week. Daily food and water intake were measured to determine metabolic state.

### 2.7. Sample collection and biochemical analysis

At the time of study termination (day 60), animals were fasted overnight (12 h, with water provided *ad libitum*) and anaesthetised with diethyl ether by inhalation in a closed chamber [43]. Blood samples (3–4 ml) were taken via cardiac puncture in non-anticoagulant-containing tubes, centrifuged at 3000 rpm at room temperature for 15 min, and the clot was removed to obtain serum. We kept the serum samples at -80°C until the biochemical analysis [43].

The fasting blood glucose value was determined with a glucometer (Accu-Chek Active, Roche Diagnostics, Germany) from sample of the tail vein puncture [33]. To obtain an indicator of glucose metabolism control, we measured glycated hemoglobin (HbA1c) concentration in blood using a boronate affinity chromatography technique (Nycocard HbA1c Test Kit, Abbott Diagnostics, USA), according to the manufacturer’s guidelines [44]. The results were expressed as % total hemoglobin (%).

We used the Rat Cystatin C ELISA Kit (MyBioSource, Inc., San Diego, CA, USA) according to the manufacturer’s standard protocol to assess the serum Cystatin C levels. The results are expressed in mg/l.

Serum SOD activity was measured by a spectrophotometric method in which superoxide is generated by the xanthine/xanthine oxidase system and detected as inhibition of cytochrome c reduction [11, 12]. We used the Superoxide Dismutase Assay Kit (Cayman Chemical, Ann Arbor, MI, USA) to assess SOD activity, according to the manufacturer’s recommendations. Results are given in U/ml.

### 2.8. Histopathological examination

After blood collection, both kidneys were rapidly removed, weighed, and fixed in 10% neutral buffered formalin for 24 h [45]. Left kidneys’ tissues were dehydrated by passage through an escalating alcohol series, followed by a paraffin infiltration process, and sections were made with 5 μm thickness, subjected to hematoxylin and eosin (H&E) staining [45]. A blinded method was used. A pathologist was employed to perform a histopathological assessment on each group. The process was performed using an Olympus CX22 LED binocular light microscope (Olympus Corporation, Japan), equipped with the Vivo 9 camera for image capture. Slides from the kidney were observed at 100x and 200x magnification. Non-overlapping, at least 10 fields per section were examined for the following criteria using a semi-quantitative scoring system adapted from established renal injury scoring methods [46]. Glomerular changes (hypertrophy, sclerosis), tubular alterations (degeneration, necrosis, and cast formation), vascular congestion, and interstitial inflammation were semi-quantitatively scored as: 0 (absent), 1 (mild), 2 (moderate), or 3 (severe) [46]. The total kidney injury score (ranging from 0 to 15) was calculated as the cumulative sum of all individual parameter scores.

### 2.9. Statistical analysis

Data were organized and analyzed using SPSS version 26.0 (IBM Corporation, USA). The Shapiro-Wilk test was also used to assess data normality [47]. For some tests, the actual value was determined; our data are expressed as mean ± SD and were analyzed using a parametric test (one-way ANOVA) followed by Tukey’s post hoc test [47]. Low standard deviation (SD) values reflect controlled housing conditions and validated commercial assays, consistent with prior studies [11, 12]. Statistical analysis was carried out using the Kruskal-Wallis test and the Mann-Whitney U test when the data were not parametric. A *p-*value of < 0.05 was considered statistically significant.

## 3. Results

### 3.1. Animal survival and body weight changes

Animals were monitored daily after the alloxan injection (day 29) for hypoglycemic signs (lethargy, tremor, and tail glucose < 40 mg/dl). All animals in Groups 1 and 4 survived until the end of the experiments (*n* = 6/group). Single deaths occurred in Group-2 and Group-3, 1 rat each did not survive after an HFD-alloxan induction period (between day 30–33), due to acute alloxan-induced hypoglycemia; necropsy confirmed no infection or organ failure. Leaving finally 5 animals in each of these groups. The total survival rate was 91.7% (22/24 animals). No survival curve was generated as deaths occurred pre-treatment during the acute induction phase.

Body weights at baseline and after a HFD-alloxan induction are shown in Table 1. At baseline (day 7, prior to the induction), there were no significant differences in body weight between groups (*p* = 0.386, one-way ANOVA), indicating effective randomization. After a high-fat diet combined with alloxan injection, there were significant within-group increments of body weights in Group-2 and Group-3 by averages of 7.10 ± 4.82 gm (*p* = 0.019) and 1.46 ± 1.06 gm (*p* = 0.040), respectively. In participants assigned to Group-1 and Group-4, there were no significant weight changes (*p* = 0.070 and *p* = 0.097, respectively). At day 60 (end of treatment), Group-2 showed the greatest body weight change (176.54 ± 11.82 gm); however, inter-group comparisons at this time point did not reach statistical significance (*p* = 0.138; one-way ANOVA).

**Table 1.** Comparison of average body weight before induction and at the end of the experiment.

Groups	Before induction (gm)	End of the experiment (gm)	*p* value
Group-1 (*n* = 6)	173.13 ± 19.59	173.63 ± 19.23	0.070
Group-2 (*n* = 5)	169.44 ± 14.40	176.54 ± 11.82	0.019*
Group-3 (*n* = 5)	177.78 ± 6.52	179.24 ± 6.74	0.040*
Group-4 (*n* = 6)	162.88 ± 5.29	163.95 ± 5.25	0.097

Data expressed as mean ± SD (standard deviation). Statistical test using one-way ANOVA (analysis of variance). *p < 0.05. Group-1 and Group-4 each consisted of 6 rats; Group-2 and Group-3 each consisted of 5 rats.

### 3.2. Glycemic parameters

Biochemical analysis on day 60, at the end of the experiment, is shown in Table 2. An HFD-alloxan induction led to metabolic disturbances compared with control animals. This led to a substantial elevation in blood glucose concentration (272.40 ± 6.66 mg/dl vs 93.33 ± 8.04 mg/dl, *p* < 0.001). Also, levels of HbA1c were significantly increased in Group-2 (10.96 ± 0.36%) compared to control animals (5.17 ± 0.19%, *p* < 0.001). The blood glucose in Group-3 treated with CCE (197.20 ± 3.03 mg/dl) was significantly decreased compared with Group-2, and HbA1c levels decreased by 8.50 ± 0.15% (*p* < 0.001 vs Group-2). Moreover, the antidiabetic activity, as evidenced by blood glucose (147.83 ± 5.49 mg/dl) and HbA1c (6.58 ± 0.28%), was significantly superior with the combination of CCE and pioglitazone (Group-4) compared to Group-3 (*p* < 0.01).

**Table 2.** Biochemical parameters among all groups at the end of the experiment.

Groups	Blood Glucose (mg/dl)	*p* value	HbA1c (%)	*p* value	Cystatin C (mg/l)	*p* value	SOD (U/ml)	*p* value
Group-1 (*n* = 6)	93.33 ± 8.04	0.000*	5.17 ± 0.19	0.000*	0.34 ± 0.02	0.000*	27.63 ± 0.64	0.000*
Group-2 (*n* = 5)	272.40 ± 6.66		10.96 ± 0.36		0.44 ± 0.02		20.86 ± 1.38	
Group-3 (*n* = 5)	197.20 ± 3.03		8.50 ± 0.15		0.36 ± 0.01		25.98 ± 0.69	
Group-4 (*n* = 6)	147.83 ± 5.49		6.58 ± 0.28		0.35 ± 0.01		26.60 ± 0.49	

Data expressed as mean ± SD (standard deviation). Statistical test using one-way ANOVA (analysis of variance). Group-1 and Group-4 each consisted of 6 rats; Group-2 and Group-3 each consisted of 5 rats. *p < 0.05.

Post-hoc power analysis (G*Power v3.1.9.7, *α* = 0.05) confirmed adequate power (> 0.80) for primary endpoints comparing treatment groups to the diabetic/nephrotoxic model (cystatin C and SOD Group-3 vs Group-2). However, the SOD comparison between Group-3 and Group-4 was underpowered, consistent with the non-significant result (*p* = 0.626; Table 5).

### 3.3. Serum cystatin C levels

Serum Cystatin C represents a 29.4% increase (*p* < 0.001, one-way ANOVA; Table 2) in diabetic/nephrotoxic animals (Group-2: 0.44 ± 0.02 mg/l) compared to control (Group-1: 0.34 ± 0.02 mg/l). This elevation indicates impaired glomerular filtration and early kidney injury in the HFD-alloxan induced diabetic/nephrotoxic model.

Treatment with CCE (Group-3) significantly decreased the serum Cystatin C levels to 0.36 ± 0.01 mg/l that exhibited a 18.2% reduction in comparison to Group-2 (*p* < 0.001, Tukey’s posthoc test; Table 3). Importantly, Cystatin C levels in Group-3 were not significantly different from the control group (0.36 ± 0.01 vs. 0.34 ± 0.02 mg/l, *p* = 0.169), indicating marked attenuation of early kidney injury.

**Table 3.** Post hoc analysis comparison of Cystatin C levels between groups.

95% CI				
Group comparison	Mean difference	Minimum	Maximum	*p* value
Group-1 vs Group-2	–0.089	–0.112	–0.067	0.000*
Group-1 vs Group-3	–0.017	–0.039	0.052	0.169
Group-1 vs Group-4	–0.008	–0.029	0.013	0.697
Group-2 vs Group-3	0.072	0.048	0.095	0.000*
Group-2 vs Group-4	0.081	0.058	0.104	0.000*
Group-3 vs Group-4	0.009	–0.014	0.032	0.677

Post hoc Tukey. Group-1 and Group-4 each consisted of 6 rats; Group-2 and Group-3 each consisted of 5 rats. *p < 0.05.

In Group-4 (CCE plus pioglitazone), we had indeed observed a comparable kidney protective action with a mean Cystatin C level of 0.35 ± 0.01 mg/l, which was significantly lower by 20.5% compared to Group-2 (*p* < 0.001), but no difference was found between Group-3 (0.35 ± 0.01 vs. 0.36 ± 0.01 mg/l, *p* = 0.677). It showed comparable biochemical trends.

### 3.4. Superoxide dismutase activity

In Table 2, we found a marked reduction in the level of serum SOD was observed in Group-2 (20.86 ± 1.38 U/ml), showing 24.5% (*p* < 0.05; one-way ANOVA) as compared with Group-1 (27.63 ± 0.64 U/ml)). This depletion is indicative of excessive oxidative stress and compromised antioxidant defense in the diabetic/nephrotoxic group.

Significant recovery in SOD, reaching 25.98 ± 0.69 U/ml (24.5%) higher than the untreated NASH group (Group-2), was achieved after treatment with CCE (Group-3; *p* < 0.001; Tukey post-test). It was corresponding to the level of the control group (94.0% reversion). However, while SOD activity in Group-3 remained lower than in Group-4, the degree of normalisation observed was clinically meaningful, indicating an improvement in antioxidant defenses.

Table 4 showed that SOD activity in the combined-treated (Group-4) was significantly higher than in Group-2 (*p* < 0.001); however, there was a 96.3% recovery relative to the control (Group-1). There was no significant SOD activity difference in Group-4 compared to control (*p* = 0.184), which indicates a near control level. Group-3 and Group-4 also did not show a significant difference in SOD activity (25.98 ± 0.69 vs 26.60 ± 0.49 U/ml, *p* = 0.626), suggesting that CCE can restore antioxidant capacity.

**Table 4.** Post hoc analysis comparison of serum SOD levels between groups.

95% CI				
Group comparison	Mean difference	Minimum	Maximum	*p* value
Group-1 vs Group-2	6.773	5.331	8.216	0.000*
Group-1 vs Group-3	1.653	0.211	3.096	0.022*
Group-1 vs Group-4	1.033	–0.342	2.409	0.184
Group-2 vs Group-3	–5.120	–6.627	–3.613	0.000*
Group-2 vs Group-4	–5.740	–7.183	–4.297	0.000*
Group-3 vs Group-4	–0.620	–2.063	0.823	0.626

Post hoc Tukey. Group-1 and Group-4 each consisted of 6 rats; Group-2 and Group-3 each consisted of 5 rats. *p < 0.05.

**Table 5.** *Post-ho*c power analysis.

Comparison	Effect Size	Power	*p* value	Interpretation
Cystatin C (Group-3 vs Group-2)	1.20	0.95	< 0.001	Adequate power
SOD (Group-3 vs Group-2)	1.05	0.92	< 0.001	Adequate power
SOD (Group-3 vs Group-4)	0.45	0.62	0.626	Underpowered; exploratory trend

Effect sizes were derived from the mean ± SD values in Tables 2–4, and post-hoc power < 0.80 was interpreted as an underpowered comparison that should be considered an exploratory finding rather than definitive evidence. (G*Power v3.1.9.7, *α* = 0.05, *n* = 5–6 per group); ANOVA repeated measures, within factors). *p < 0.05.

### 3.5. Histopathological findings

**Figure 1.**
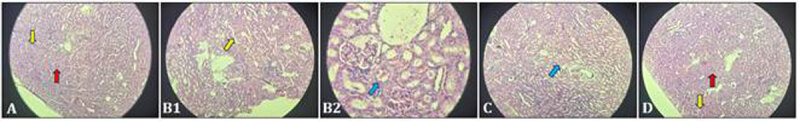
Representative microscopic images of the kidneys’ experimental groups. (H&E staining; magnification 100x in A, B1, and D; magnification 200x in B2 and C). A. Group-1; B1. Group-2; B2. Group-2; C. Group-3; D. Group-4; Red arrow: Glomerulus; Yellow arrow: Renal tubules; Blue arrow: Glomerular perihilar capillaries (Group-2 in B2), Renal tubules (Group-3 in C).

Typical micrographs of kidney sections stained with H&E are shown in Figure 1. Histopathological analysis clearly showed differences in morphological changes between the groups tested, as shown in Table 6.

**Table 6.** Semi-Quantitative Histopathological Scoring of Kidney Injury.

Parameters	Group-1 (Control)	Group-2 (Diabetic/nephrotoxic)	Group-3 (Diabetic/nephrotoxic + CCE)	Group-4 (Diabetic/nephrotoxic + CCE + Pio)	*p* value
Glomerular hypertrophy	0.17 ± 0.41	2.00 ± 0.55	1.00 ± 0.45	0.67 ± 0.52	< 0.001
Tubular dilation	0.00 ± 0.00	2.20 ± 0.55	1.00 ± 0.71	0.67 ± 0.52	< 0.001
Vascular congestion	0.17 ± 0.41	2.20 ± 0.55	1.00 ± 0.45	0.50 ± 0.41	< 0.001
Inflammatory infiltration	0.17 ± 0.41	2.00 ± 0.45	0.80 ± 0.45	0.50 ± 0.55	< 0.001
Glomerular sclerosis	0.00 ± 0.00	2.20 ± 0.55	1.00 ± 0.71	0.67 ± 0.52	< 0.001
Composite injury score	0.51 ± 0.54	10.60 ± 1.75	4.80 ± 1.76	3.01 ± 1.52	< 0.001

Data expressed as mean ± SD. Scoring: 0 = none, 1 = mild, 2 = moderate, 3 = severe. Statistical analysis using Kruskal-Wallis test followed by Mann-Whitney U test with Bonferroni correction. *p* value represents overall group comparison. All treatment groups (Group-3 and Group-4) showed significant improvement compared to Group-2 (*p* < 0.001 for all parameters). CCE: capsule combination extract; Pio: pioglitazone.

Group-1: The red arrow indicates a glomerulus, a kidney filter. It exhibited a regular size and cellularity, with intact Bowman’s capsule and capillary tufts. The yellow arrow indicates the renal tubules, which reabsorb water and solutes. Kidney sections displayed normal or mild changes of histological architecture (Figure 1A). The mean composite kidney injury score was 0.51 ± 0.54.

Group 2: The yellow arrow indicates a dilation in the renal tubule with some degeneration or accumulation of cells. A protein cast of cellular debris fills a dilated renal tubule. The surrounding tubules demonstrate degeneration or necrosis, indicative of renal injury. Severe pathological changes were observed in kidney sections (Figures 1B1 & 1B2). Important abnormalities included: - Glomerular hypertrophy: Enlarged glomeruli with a hypercellularity and an expansion of the mesangial matrix (score 2.00 ± 0.55) - Tubular changes: Extensive tubular dilation, epithelial flattening, brush border loss, cytoplasmic vacuolization (score 2.20 ± 0.55) - Vascular abnormalities: A focal dilation of afferent arterioles associated to a strong perihilar glomerular capillaries (Blue arrow) congestion (score 2.20 ± 0.55) - Inflammatory infiltration: Moderate to severe interstitial infiltration characterized mainly by lymphocytes and macrophages (score 2.00 ± 0.45) - Glomerular sclerosis: segmental perihilar sclerosis consisting in an increase of collagen deposition and capillary collapses (score 2.20 ± 0.55). The mean composite kidney injury score was 10.60 ± 1.75 and was significantly higher than in the control (*p* < 0.001, Kruskal-Wallis).

Group-3: The blue arrow (Figure 1C) indicates a renal tubule that looks dilated/changed, but possibly less extreme than observed in Figure 1B1. However, CCE treatment evidently improved histopathological outcomes. Key histological findings compared to Group-2 were: - attenuation of glomerular hypertrophy with normalization of size and cellularity (score: 1.00 ± 0.45 vs. 2.00 ± 0.55, *p* < 0.001) - reduction in tubular dilation with restitution to integrity of the epithelium in most areas (score: 1.00 ± 0.71 vs, 2.20 ± 0.55; *p* < 0,001) - marked reduction in vascular congestion (score: 1.00 ± 0.45 vs. 2.20 ± 0.55; *p* < 0.001) -minimal inflammatory cell infiltration (score: 0.80 ± 0.45 vs. 2.00 ± 0.45; *p* < 0.001) - Diminished glomerular sclerosis with preservation of capillary architecture (score: 1.00 ± 0.71 vs. 2.20 ± 0.55, *p* < 0.001). The mean composite kidney injury score was 4.80 ± 1.76, or a 54.7% decrease compared with Group-2 (*p* < 0.001). Although mild residual abnormalities remained, semi-quantitative scoring showed marked attenuation compared with the model (54.7% reduction in composite score, *p* < 0.001). The inflammatory infiltration score showed no significant difference compared with the control (*p* = 0.127). Future morphometry suggested.

Group 4: A red arrow indicates a glomerulus, and a yellow arrow indicates renal tubules (Figure 1D). The combination treatment resulted in the best histopathological recovery compared to Group-2 (Figures 1 B1, B2): - Marked further improvement in glomerular hypertrophy (score: 0.67 ± 0.52 vs. 2.00 ± 0.55; *p* < 0.001) - Tubular dilation, minimal with well-preserved epithelial cytology (score: 0.67 ± 0.52 vs. 2.20 ± 0.55; *p* < 0.001) - Absent to minimal vascular congestion (score: 0.50 ± 0.41 vs. 2.20 ± 0.55; *p* < 0.001) - Negligible inflammatory infiltration (score: 0.50 ± 0.55 vs. 2.00 ± 0.45; *p* < 0.001) - Minimal sclerotic changes (score: 0.67 ± 0.52 vs. 2.20 ± 0.55; *p* < 0.001). The mean composite kidney injury score was 3.01 ± 1.52, which was significantly decreased by 71.6% compared to Group-2 (*p* < 0.001). Notably, Group-4 had significantly lower injury scores than Group-3 for glomerular hypertrophy (*p* = 0.041), tubular dilation (*p* = 0.032), and the composite score (*p* = 0.048), suggesting that pioglitazone provided additional histological benefit.

## 4. Discussion

Our model ultimately succeeded in inducing hyperglycemia, elevated HbA1c, and characteristic metabolic perturbations, confirming the induction of kidney injury under NASH-like metabolic conditions closely resembling human NASH pathophysiology [31, 32]. This study used HFD + alloxan to induce hyperglycemia and systemic oxidative stress [31, 32].

However, hepatic NASH pathology (steatosis, inflammation, fibrosis) was not confirmed histologically; therefore, kidney injury likely stems from multifactorial mechanisms-diabetes-induced glomerulosclerosis and oxidative stress combined with direct alloxan tubular toxicity as a confounder rather than pure NASH-mediated kidney injury [33, 34]. Although this model does not provide direct histological confirmation of hepatic NASH, the metabolic and oxidative stress milieu it produces allows us to explore nephroprotective effects relevant to kidney complications frequently observed in patients with NASH and related metabolic disorders [5, 6]. This model limitation precludes clear separation of NASH-specific vs diabetic/toxic mechanisms. Future studies should include liver histology or employ methionine-choline-deficient (MCD) diet models for NASH specificity [48].

This study presents new findings that the standardized combination of *Channa striata, Phyllanthus niruri* L., and *Curcuma xanthorrhiza* extracts successfully ameliorates kidney injury in HFD-alloxan-induced diabetic nephrotoxicity through multiple complementary mechanisms [25, 26, 27, 28, 29, 30]. It demonstrated a marked attenuation of early injury biomarkers, re-establishment of antioxidant defense, and preservation of renal histological structure.

The higher serum Cystatin C levels in HFD-alloxan-induced diabetic/nephrotoxic animals indicate early kidney injury, which is in line with its occurrence in 20–40% of human patients, as reported by Sandireddy et al. [5] and Kanbay et al. [6]. The 29.4% upregulation of Cystatin C in our HFD-alloxan-induced diabetic/nephrotoxic model is supported by clinical findings showing higher Cystatin C levels in patients with NASH and demonstrates the validity of our experimental approach [15, 16].

The decrease in SOD activity in diabetic-nephrotoxic animals (24.5%) also provided evidence of the involvement of oxidative stress in the etiology of liver injury [9, 10, 11, 12]. SOD is the first line of enzymatic antioxidant defense, mediating the dismutation of superoxide radicals to hydrogen peroxide and oxygen [11, 12]. In NASH, oversufficient ROS production caused by mitochondrial dysfunction, cytochrome P450 2E1 activation, and inflammatory cell infiltration would overwhelm SOD capacity, leading to lipid peroxidation, protein carbonylation, and cellular damage [7, 8, 9, 10]. This oxidative burden is widespread, involving renal tissue via the same pathways as systemic issues, as demonstrated by our kidney histopathology, which showed changes particularly in the glomerulus and tubules [5, 6, 7, 8, 10].

Treatment with CCE was beneficial in correcting these pathophysiological changes [25, 26, 27, 28, 29, 30]. Cystatin C was reduced by 18.2%, and SOD activity was restored to 94% of control levels, indicating that the herbal combination acts on a primary disease mechanism rather than merely on symptomatic manifestations. The nephroprotective properties are probably due to the synergistic effects of these compounds [22, 23, 24]. Research conducted by Abd Hadi et al. [25] and Sasongko et al. [26] confirmed that *Channa striata* contains albumin with essential amino acids that aid tissue repair and cellular regeneration, along with antioxidant peptides [25, 26]. *Phyllanthus niruri* L. has lignans (phyllanthin, hypophyllanthin), and flavonoids as free radical scavengers and up-regulates the expressions of endogenous antioxidant enzymes by activating Nuclear Factor Erythroid 2-related factor 2 (*Nrf2*) pathway and suppresses pro-inflammatory cytokines [27, 28]. *Curcuma xanthorrhiza* possesses curcuminoids with Nuclear Factor-Kappa B (*NF-κB*) inhibitory activity. It also decreases oxidative stress markers and enhances mitochondrial function [29, 30].

The non-significant difference between CCE alone and combination treatment with CCE and pioglitazone in renal outcomes merits a comment. Although combination CCE treatment has better antidiabetic effects, the renal protection indices did not differ significantly between mono- and combination-treatments, indicating that common CCE’s nephroprotective mechanisms are independent of glucose-lowering activity [18, 19, 20, 25, 26, 27, 28, 29, 30]. This result is consistent with data from some references, which highlighted that oxidative stress and inflammation were involved in kidney injury under NASH-like metabolic conditions, via a mechanism beyond hyperglycemia itself [5, 6, 7, 8]. The peroxisome proliferator-activated receptor gamma (PPAR-*γ*) agonist, pioglitazone, also improves insulin action and attenuates hepatic inflammation but may lead to weight gain, fluid retention, and cardiovascular complications [18, 19, 20]. The pioglitazone dose of 1.35 mg/kg BW (~0.27 mg/200 gm BW) was intentionally low for chronic 30-day administration to mimic human therapeutic exposure while minimizing potential side effects such as fluid retention or weight gain observed at higher doses [18, 19, 20]. Rodent literature reports a wide range of pioglitazone doses, from 0.3 mg/kg (nephroprotection in the gentamicin model) to 60 mg/kg (adenine CKD) [42], with 1–10 mg/kg most commonly used in diabetes and kidney injury models [40, 41]. While some studies employ higher acute doses (10–30 mg/kg), our chronic regimen aligns with translational approaches prioritizing safety and relevance to clinical practice [41, 42].

Nonetheless, the absence of a pioglitazone-only group limits direct comparison of CCE additive effects, representing an avenue for future investigation. Our results may indicate that CCE could be an alternative and a safe option in treating renal complications of NASH-like metabolic condition patients, particularly for individuals who are intolerant or unable to receive pioglitazone therapy [21, 22].

These biochemical results were supported by corresponding histopathological changes, including preserved glomerular architecture, less tubular damage, and minimal vascular congestion in CCE-treated animals [25, 26, 27, 28, 29, 30, 45, 46]. These architectural changes convey the translation of biochemical nephroprotection into a tissue-level phenomenon critical to long-term kidney function salvage. The 54.7% reduction in histopathological severity scores with CCE treatment indicates substantial disease modification rather than mere biomarker modulation [25, 26, 27, 28, 29, 30]. This comparison (Group–3 vs. Group–4) established the fact that CCE by itself (Group–3), rendered significant protection to the kidney and hence has no dependency on any additional pharmacological intervention.

The clinical relevance of our results is important. Given the absence of approved therapies specifically targeting kidney injury in a HFD-alloxan induced diabetic/nephrotoxic or NASH-like metabolic conditions, CCE is a potential novel agent that deserves further clinical exploration [21, 22]. The use of chemically defined and standardized herb extracts could be considered a way to address concerns about low quality control and reproducibility in herbal medicine [49, 50]. Moreover, the good tolerability of bioactive herb extracts used in traditional medicine and preclinical studies suggests clinical applicability [22, 23, 24, 49, 50].

Several limitations should be acknowledged. First, our study was short-term (60 days) and would need to be followed by a longer-term study to determine sustained efficacy and any potential disease-modifying effect [51]. Second, although we showed SOD improvement and a decrease in Cystatin C, a more comprehensive assessment of other oxidative stress markers (malondialdehyde, glutathione, catalase) and inflammatory mediators would provide further mechanistic data [7, 8, 9, 10]. Third, while our NASH model has been used extensively, it still may not completely mimic certain features of human pathophysiology of NASH, including chronic progressive disorder [31, 32]. Fourth, the single role of each herb relative to its combined effect was not evaluated; therefore, whether the actions are synergistic or simply additive remains a question [22, 23, 24]. Fifth, only cystatin C and SOD were used in this study to evaluate renal injury and oxidative stress. Future studies should expand biomarker panels to include: serum creatinine, BUN, and urinary injury markers; oxidative stress markers (malondialdehyde, glutathione, catalase, glutathione peroxidase); inflammatory cytokines (*TNF-α, IL-6*); and future quantitative morphometry (e.g., glomerular diameter). These additions would strengthen mechanistic conclusions beyond preliminary antioxidant effects. Sixth, the lack of liver biochemistry/histology precludes inferences regarding pure NASH pathophysiology, and hepatic NASH confirmation is required.

Further research should be designed to include dose-response studies for the optimal formulation of CCE, to asses the molecular mechanisms at gene expression and protein level, to find whether hepatic pathology induced by NASH can be affected as well in parallel or independently from kidney protection, and examination with other NASH models (methionine-choline-deficient diet, Western diet) would also be confirmed [51, 52, 53]. Moreover, it would be of interest to elucidate the absorption, distribution, metabolism, and excretion of essential bioactive compounds using pharmacokinetic methodologies to propose effective dosages for clinical use [54].

## 5. Conclusions

In this study, we have shown that the standardized capsule combination extract of *Channa striata, Phyllanthus niruri* L., and *Curcuma xanthorrhiza* significantly reduces kidney injury in HFD/alloxan-induced diabetic/nephrotoxic rats. The beneficial effects of CCE were observed through restoring antioxidant defenses (SOD activity), decreasing the early renal injury marker (cystatin C), and improving kidney histology. While CCE in combination with pioglitazone produced additional histological benefits, our data do not allow us to claim equivalence to pioglitazone, and the absence of a pioglitazone-only group is acknowledged as a limitation. Other limitations include alloxan confounding, absence of hepatic NASH confirmation, limited biomarkers, and a short 60-day duration. The results suggest that the CCE combination may be beneficial for kidney complications occurring in metabolic/NASH-related settings. These preliminary findings provide a scientific basis for further study of this herbal combination as a potential adjunct for the treatment of metabolic kidney complications.

## Data Availability

The data presented in this study are available from the corresponding author upon reasonable request.

## List of abbreviations

ANOVA, analysis of variance; BW, body weight; BUN, blood urea nitrogen; CCE, capsule combination extract; CKD, chronic kidney disease; ELISA, enzyme-linked immunosorbent assay; GFR, glomerular filtration rate; H&E: Hematoxylin and Eosin; HbA1c, glycated hemoglobin; HFD, high-fat diet; *IL-6*, interleukin-6; Na-CMC, Sodium Carboxymethyl cellulose; NAFLD, non-alcoholic fatty liver disease; NASH, non-alcoholic steatohepatitis; *NF-κB*, Nuclear Factor-Kappa B; *Nrf2*, Nuclear Factor Erythroid 2-Related Factor 2; PPAR-*γ*, peroxisome proliferator-activated receptor gamma; ROS, reactive oxygen species; SOD, superoxide dismutase; *TNF-α*, tumor necrosis factor-alpha; dl, deciliter; gm, gram; h, hour; kg, kilogram; l, liter; m, meter; mg, milligram; min, minute; ml, milliliter; rpm, revolutions per minute.
